# Supplementary material for: Atazanavir Is a Competitive Inhibitor of SARS-CoV-2 Mpro, Impairing Variants Replication In Vitro and In Vivo
Source: Pharmaceuticals (Basel). 2021 Dec 24;15(1):21. doi: 10.3390/ph15010021 (PMC8777605; doi:10.3390/ph15010021)
Supplement: Supplementary file 1 [file pharmaceuticals-15-00021-s001.zip › pharmaceuticals-1508186-supplementary.pdf]

Article

Atazanavir Is a Competitive Inhibitor of SARS-CoV-2 M^{pro}, Impairing Variants Replication In Vitro and In Vivo

Otávio Augusto Chaves [1,2,*,†], Carolina Q. Sacramento [1,2,†], André C. Ferreira [1,2,3,†], Mayara Mattos [1,2,†], Natalia Fintelman-Rodrigues [1,2], Jairo R. Temerozo [4,5], Leonardo Vazquez [1], Douglas Pereira Pinto [6], Gabriel P. E. da Silveira [6], Laís Bastos da Fonseca [6], Heliana Martins Pereira [6], Aluana Santana Carlos [3], Joana C. d'Avila [1,3], João P. B. Viola [7], Robson Q. Monteiro [8], Patrícia T. Bozza [1], Hugo Caire Castro-Faria-Neto [1] and Thiago Moreno L. Souza [1,2,*]

Laboratory of Immunopharmacology, Oswaldo Cruz Institute (IOC), Oswaldo Cruz Foundation (Fiocruz), Rio de Janeiro, RJ, 21040-360, Brazil; carol.qsacramento@gmail.com (C.Q.S.); andre.bio2009@gmail.com (A.C.F.); maymattos03@gmail.com (M.M.); nataliafintelman@gmail.com (N.F.-R.); leonardo_vazquez@hotmail.com (L.V.); joanacpdavila@gmail.com (J.C.d'A.); pbozza@gmail.com (P.T.B.); hugocfneto@gmail.com (H.C.C.-F.-N.)

2 National Institute for Science and Technology on Innovation on Neglected Diseases Neglected Populations (INCT/IDNP), Center for Technological Development in Health (CDTS), Oswaldo Cruz Foundation (Fiocruz), Rio de Janeiro, RJ, 21040-900, Brazil

3 Preclinical Research Laboratory, Universidade Iguaçu—UNIG, Nova Iguaçu, RJ, 26260-045, Brazil; aluanasc@gmail.com

4 Laboratory on Thymus Research, Oswaldo Cruz Institute (IOC), Oswaldo Cruz Foundation (Fiocruz), Rio de Janeiro, RJ, 21040-900, Brazil; jairo.jrt@gmail.com

5 National Institute for Science and Technology on Neuroimmunomodulation (INCT/NIM), Oswaldo Cruz Institute (IOC), Oswaldo Cruz Foundation (Fiocruz), Rio de Janeiro, RJ, 21040-900, Brazil

6 Laboratory of Pharmacokinetics, Vice Presidency of Research, and Innovation in Health—Oswaldo Cruz Foundation (Fiocruz), Rio de Janeiro, RJ, 21040-900, Brazil; douglas.pinto@fiocruz.br (D.P.P.); gabriel.silveira@fiocruz.br (G.P.E.d.S.); lais.fonseca@fiocruz.br (L.B.d.F.); heliana.pereira@fiocruz.br (H.M.P.)

7 Program of Immunology and Tumor Biology, Brazilian National Cancer Institute (INCA), Rua André Cavalcanti 37, 5th floor, Centro, Rio de Janeiro, RJ, 20231-050, Brazil; jpviola@inca.gov.br

8 Institute of Medical Biochemistry Leopoldo de Meis, Federal University of Rio de Janeiro, Rio de Janeiro, RJ, 21941-902, Brazil; robsonqm@bioqmed.ufrj.br

* Correspondence: otavioaugustochaves@gmail.com (O.A.C.); tmoreno@cdts.fiocruz.br (T.M.L.S.)

† These authors contributed equally to this work.

Citation: Chaves, O.A.; Sacramento, C.Q.; Ferreira, A.C.; Mattos, M.; Fintelman-Rodrigues, N.; Temerozo, J.R.; Vazquez, L.; Pinto, D.P.; da Silveira, G.P.E.; da Fonseca, L.B.; et al. Atazanavir is a Competitive Inhibitor of SARS-CoV-2 M^{pro}, Impairing Variants Replication In Vitro and In Vivo. *Pharmaceuticals* **2022**, *15*, 21. https://doi.org/10.3390/ph15010021

Academic Editor(s): Jean Jacques Vanden Eynde; Annie Mayence

Received: 29 November 2021
Accepted: 20 December 2021
Published: 24 December 2021

Publisher's Note: MDPI stays neutral with regard to jurisdictional claims in published maps and institutional affiliations.

Copyright: © 2021 by the authors. Licensee MDPI, Basel, Switzerland. This article is an open access article distributed under the terms and conditions of the Creative Commons Attribution (CC BY) license (https://creativecommons.org/licenses/by/4.0/).

Abstract: Atazanavir (ATV) has already been considered as a potential repurposing drug to 2019 coronavirus disease (COVID-19); however, there are controversial reports on its mechanism of action and effectiveness as anti-severe acute respiratory syndrome coronavirus 2 (SARS-CoV-2). Through the pre-clinical chain of experiments: enzymatic, molecular docking, cell-based and in vivo assays, it is demonstrated here that both SARS-CoV-2 B.1 lineage and variant of concern gamma are susceptible to this antiretroviral. Enzymatic assays and molecular docking calculations showed that SARS-CoV-2 main protease (M^{pro}) was inhibited by ATV, with Morrison's inhibitory constant (K_i) 1.5-fold higher than GC376 (a positive control) dependent of the catalytic water (H_2O_{cat}) content. ATV was a competitive inhibitor, increasing the M^{pro}'s Michaelis–Menten (K_m) more than sixfold. Cell-based assays indicated that different lineages of SARS-CoV-2 is susceptible to ATV. Using oral administration of ATV in mice to reach plasmatic exposure similar to humans, transgenic mice expression in human angiotensin converting enzyme 2 (K18-hACE2) were partially protected against lethal challenge with SARS-CoV-2 gamma. Moreover, less cell death and inflammation were observed in the lung from infected and treated mice. Our studies may contribute to a better comprehension of the M^{pro}/ATV interaction, which could pave the way to the development of specific inhibitors of this viral protease.

Keywords: SARS-CoV-2; COVID-19; repurposing drugs; atazanavir; protease inhibitor; pharmacokinetics; molecular docking

SUPPLEMENTARY MATERIAL

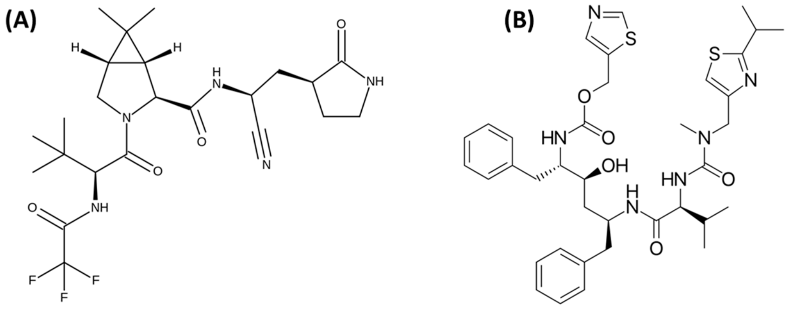

Figure S1. Chemical structure for (A) PF-07321332 and (B) ritonavir, the active principles of PAXLOVID™ from Pfizer.

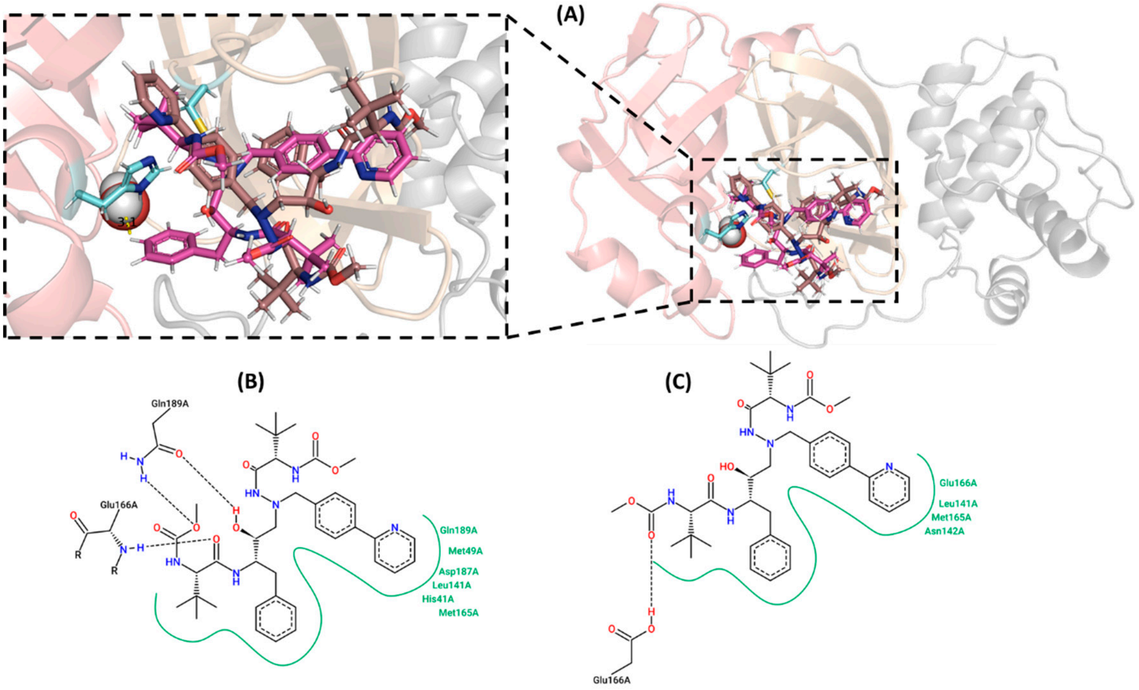

Figure S2. (A) Superposition of the best docking pose for the interaction between M^{pro} and ATV in the presence and without the catalytic water H_2O_{cat} (ATV in pink and brown, respectively). The 2D-plot image for the interaction among the amino acid residues from the catalytic pocket of M^{pro} with (B) ATV in the presence of H_2O_{cat} and (C) ATV without H_2O_{cat}. For better interpretation the M^{pro} structure was represented only in the monomeric form with the domains I, II, and III in light red, orange, and gray, respectively. The catalytic dyad His-41 and Cys-145 are represented as sticks in cyan, while the amino acid residues which interact hydrophobically with ATV are in green in the 2D-plot image.

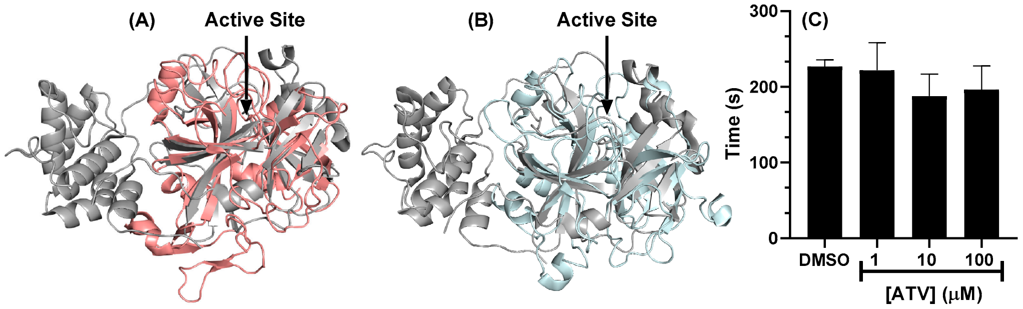

Figure S3. Superposition of the monomeric unit of M^{pro} (in gray, PDB code 7K40) with (A) FXa (in salmon, PDB code 2P16) and (B) thrombin (in cyan, PDB code 1KTS). For better interpretation the catalytic water (H_2O_{cat}) of M^{pro} is not shown. (C) Fibrin formation trial without and in the presence of three concentrations of ATV.

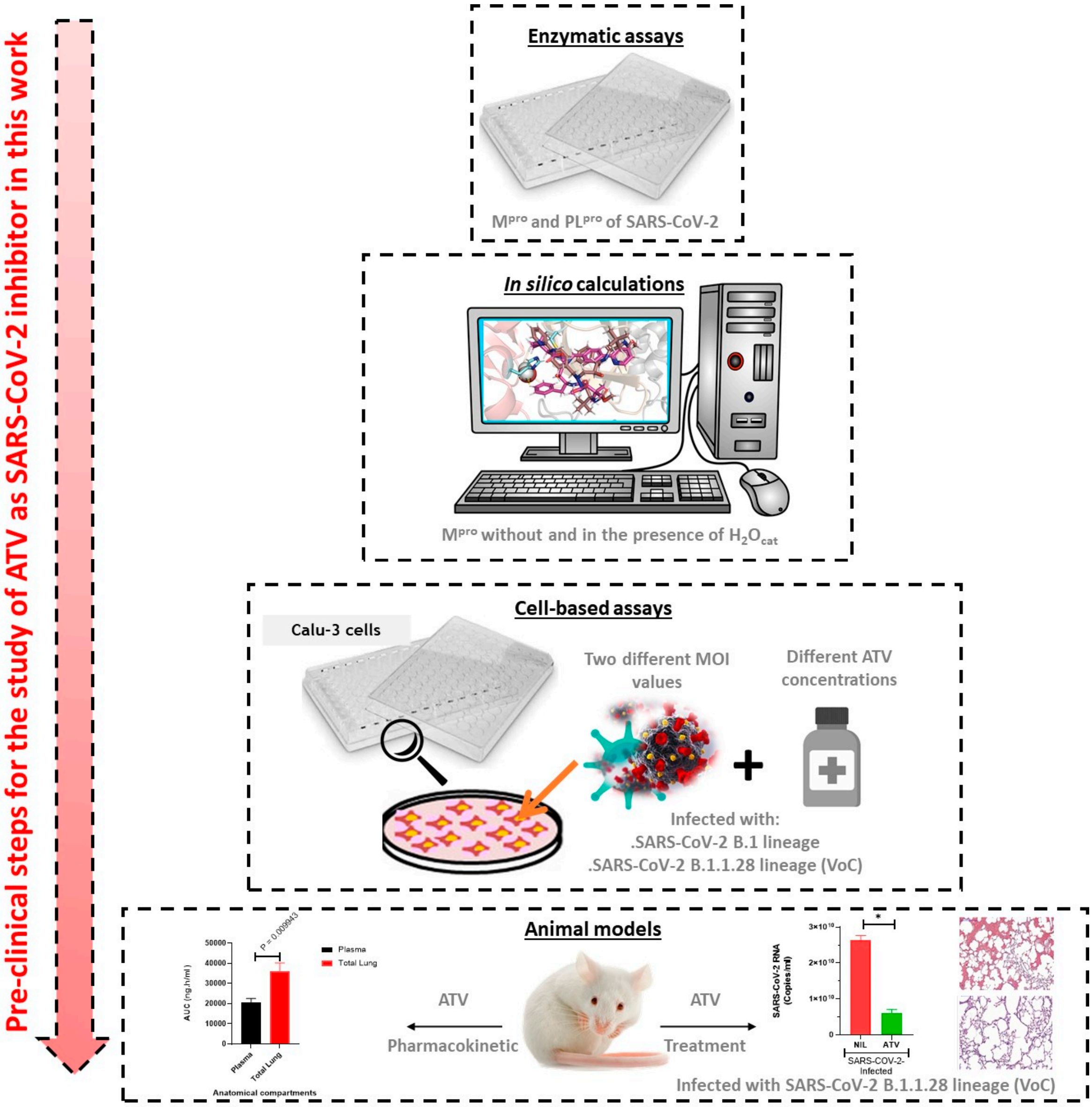

Figure S4. The flow charge indicating the main steps of the present work.
